# Whole-Genome Sequence of the C57L/J Mouse Inbred Strain

**DOI:** 10.1534/g3.114.012997

**Published:** 2014-07-21

**Authors:** Guruprasad Ananda, Yuka Takemon, Douglas Hinerfeld, Ron Korstanje

**Affiliations:** The Jackson Laboratory, Bar Harbor, Maine

**Keywords:** genome, mouse, sequence

## Abstract

We sequenced the complete genome of the widely used C57L/J mouse inbred strain. With 40× average coverage, we compared the C57L/J sequence with that of the C57BL/6J and identified many known as well as novel private variants. This genome sequence adds another strain to the growing number of mouse inbred strains with complete genome sequences and is a valuable resource to the scientific community.

The C57L/J inbred strain originates from The Jackson Laboratory and was derived from a now extinct sub-strain of C57BR after a mutation in 1933 in the *Mlph* gene, providing its distinct "leaden" coat color (Matesic *et al.* 2001). Other distinct phenotypes of this strain, which have been the subject of study in the past, are the high incidence of Hodgkin-like reticulum cell neoplasm (Levine and Sowinski 1973), susceptibility to experimental encephalomyelitis (Levine and Sowinski 1973), atherosclerosis (Nishina *et al.* 1993), and the development of gallstones (Paigen 1995).

Because of its frequent use in studies and its relatedness to C57BL/6J, sequencing of its complete genome and the identification of variants leading to altered gene function are of value to the research community.

## Materials and Methods

### Library construction and high-throughput sequencing

Purified genomic DNA from spleen of a male C57L/J mouse (stock #668; The Jackson Laboratory, Bar Harbor, ME) was fragmented using a Bioruptor [Diagenode B0101001 (UCD-200 TM-EX) Denville, NJ] two times for 10 min on low power alternating 30′′ sonication and 30′′ pause. End repair, 3′ adenylation, adapter ligation, and bead clean-up were performed using the Illumina (San Diego, CA) TruSeq DNA Sample Preparation LT kit. DNA fragments were enriched for sequencing per the TruSeq method with the following modifications: 18 cycles of PCR with NEB (Ipswitch, MA) Phusion HF and TruSeq oligos at 2 uM final concentration in a 100-ul reaction. After bead clean-up, the library was size-selected for 320–450 bp on a Pippin Prep (Sage Science, Beverly, MA). The final library was quantified by qPCR and sequenced on three lanes on an Illumina HiSequation 2000 using the 2× 100 paired end method.

### Sequence Analysis

#### Read quality assessment and mapping:

Reads were quality trimmed and filtered using the NGS QC toolkit v2.3 (Patel and Jain 2012) to remove reads containing more than 30% low-quality (Q < 30) bases. The resulting high-quality reads were aligned to the December 2011 release of the mouse reference genome (mm10) from UCSC using BWA v0.5.10 (Li and Durbin 2009). Duplicates were removed using Picard v1.95 (http://picard.sourceforge.net) and the alignments were preprocessed (including realignments around indels and base quality score recalibration).

#### SNP and indel calling:

SNPs and indels were called using the GATK tool suite v2.7.4 (McKenna *et al.* 2010; Depristo *et al.* 2011). The variants that met the following criteria were flagged as potential artifacts in the VCF file: (1) coverage < 5; (2) variant quality < 50; (3) strand bias Phred-scaled p-value > −10; (4) at least four reads with mapping quality of 0 and more than 10% of the aligned reads map ambiguously; and (5) present in tandem repeats longer than 10-bp with motif size < 7. Finally, genomic and functional annotations were assigned to the variants using SnpEff v2.0.5 (Cingolani *et al.* 2012). Each variant was assigned one of the four types of impacts based on how significant the effect of the variant is: high (*e.g.*, frame shift, stop gain/loss, start loss, etc.); moderate (*e.g.*, nonsynonymous coding changes, codon insertion/deletion, etc.); low (*e.g.*, synonymous changes etc.); or modifier (*e.g.*, changes up/downstream of coding regions, intronic changes, intergenic changes, etc.).

#### Structural variant (SV) calling:

Using the mapped reads, structural variants (insertions, deletions, and inversions) were called using BreakDancerMax (Chen *et al.* 2009) and Pindel (Ye *et al.* 2009). The two call-sets were merged using SVmerge (Wong *et al.* 2010) and only SVs that were at least 100-bp long were retained.

### Genotyping using the MegaMUGA

DNA from the same mouse was submitted to GeneSeek (Lincoln, NE) for hybridization on the Mega Mouse Universal Genotyping Array (MegaMUGA), which provides 77,800 SNP markers built on the Illumina Infinium platform. Data were analyzed using BEDtools v2.17.0 (Quinlan and Hall 2010).

#### Data accession code:

The BAM files (containing aligned and unaligned reads) can be accessed from the NCBI Sequence Read Archive (SRA) using the following SRA accession code: SRS635099.

## Results

### Alignment and coverage

Sequencing of the C57L/J resulted in 1,744,197,122 reads, of which 1,237,576,596 (71%) were considered of high enough quality for alignment. Reads were aligned to the published C57BL/6 genome [December 2011 release of the mouse reference genome (mm10) from UCSC]. Approximately 95.8% of the reference genome was covered by at least five reads, with a mean genome-wide coverage of 39.2× (mean coverages on autosomes and sex chromosomes are 40.9× and 23.3×, respectively). The sequence reads (in fastq format), alignments (in BAM format), and variant calls (in VCF format) can be accessed through mousegenomes.jax.org

### Identification of variants in the C57L/J genome

In the C57L/J genome 2,385,932 single nucleotide polymorphisms (SNPs), 419,568 indels (small insertions and deletions), and 7091 SVs (structural variants) were called using the GATK tool suite and SVmerge (see *Materials and Methods*). These variants were compared with variant calls from 18 key mouse strains from the Sanger Institute (Keane *et al.* 2011) as well as variants from male and female C57BL/6J genomes (sequenced at The Jackson Laboratory). This led to the identification of 198,864 (8.3%) SNPs, 52,693 (12.6%) indels, and 807 (11.4%) SVs that were unique to the C57L/J genome. The MegaMUGA provided data for 75,890 markers, of which 63,605 were called the same as the mm10 reference and 12,285 were different; 73,962 of these markers were in agreement with our variant calls, indicating high genotyping accuracy (97.5%) of our variant set.

### Early stop codons and frame shifts

We categorized the identified intra-genic SNPs and indels as high (0.05%), moderate (0.38%), and low (0.32%) impact and focused on the 114 variants with high impact that were unique to C57L/J. These are variants that lead to splice site changes, frame shifts, loss of the start or stop site, and the gain of early stop codons. Among these is the SNP in *Mlph* (p.R31*) that leads to an early stop codon and gives C57L/J its distinct coat color. We performed Sanger sequencing on all 114 variants. Our Sanger sequencing of the high-impact variants gives a good indication of the false discovery rate. Of the 69 variants with an allele frequency below 0.8 that we tested, 12 were confirmed (FDR = 0.83) and the others were false positives. Among the 45 variants with an allele frequency of 1.0, 36 were confirmed (FDR = 0.20). The data for the confirmed high impact variants are summarized in Table 1.

**Table 1:** Confirmed high impact variants unique to C57L/J

Chr	Position	Gene	B6	C57L	Effect
1	11588478	*A830018L16Rik*	GC	G	Frameshift
1	78967942	*Gm5830*	TGTAA	T	Frameshift
1	90921959	*Mlph*	C	T	R31*
1	161083474	*Cenpl*			*330Y
1	182157736	*Vmn1r1*	CA	C	Frameshift
2	154234491	*Bpifb5*	C	T	Q396*
3	28604342	*Tnik*	T	A	Splice site
4	111939648	*Skint8*	G	A	W316*
4	111981948	*Skint7*	GACAGAGAT	G	Frameshift
4	112298168	*Skint3*	AGAGAATAT	A	Frameshift
4	113479673	*Skint5*	GT	G	Frameshift
4	113479676	*Skint5*	A	AT	Frameshift
4	144362992	*Gm13119*	G	A	Splice site
4	147228756	*AL731663.2*	T	G	Splice site
5	90243042	*Ankrd17*	G	GT	Frameshift
5	90243443	*Ankrd17*	G	A	Q1872*
6	50846755	*G930045G22Rik*	C	T	Splice site
6	129874907	*Klra17*	T	C	M1V
7	8244983	*Vmn2r43*	G	T	S727*
7	8367484	*Vmn2r44*	CT	C	Frameshift
7	67660681	*Ttc23*	AG	A	Frameshift
7	131074205	*Dmbt1*	AACAGAAAACA	GACAGATTCTG	E597D, N598S, S599G
8	58912859	*BC030500*	G	A	Splice site
8	58913115	*BC030500*	G	A	W158*
8	63928657	*Gm4975*	AT	A	Frameshift
9	3037490	*Gm10715*	G	T	Splice site
10	129540729	*Olfr792*	CTCA	TGCT	S65A
11	59055475	*Obscn*	GCCTG	AACCC	P3845T,V3846L
11	65152580	*1700086D15Rik*	T	TCAACA	Frameshift
11	67709643	*Glp2r*	AC	A	Frameshift
11	67709646	*Glp2r*	C	CG	Frameshift
12	103868760	*Gm8895*	C	T	Splice site
13	14193478	*Arid4b*	GTA	ACG	Frameshift
13	31408776	*Gm11375*	A	ACC	Frameshift
14	31001359	*Spcs1*	GC	G	Frameshift
14	32806908	*1810011H11Rik*	CAGGGTA	C	Frameshift
14	69776182	*Tnfrsf10b*	TC	T	Frameshift
15	5120767	*Gm10250*	CCTCA	C	Frameshift
15	76296701	*Oplah*	CG	C	Frameshift
17	35266714	*H2-D1*	C	T	R358*
17	36190120	*H2-T3*	C	T	M1I
17	47378266	*Mrps10*	AGG	A	Frameshift
17	63863369	*A930002H24Rik*	G	T	C141*
17	63863679	*A930002H24Rik*	CG	C	Frameshift
19	7639603	*Hrasls5*	C	T	Q328*
19	11459243	*Ms4a4b*	GGTGTAG	AATATAA	G126N, V127I, V128I
19	13861863	*Olfr1502*	G	A	W23*
X	137015306	Slc25a53	ACAGCGGCACATGGCTCCCACAGCAGG	A	Frameshift

## Discussion

Because the C57L/J strain is used regularly in mapping of quantitative traits like physical activity (Leamy *et al.* 2010), obesity (Taylor and Phillips 1997), and gallstones (Paigen *et al.* 2000), as well as a mapping strain for ENU mutants (Aljakna *et al.* 2012), obtaining the full genome sequence and a comparison with the related C57BL/6J is beneficial to the research community. It provides SNPs for denser genetic mapping as well as the rapid identification of possible causal variants in candidate genes.

We sequenced the genome of a male C57L/J mouse and, subsequently, compared the sequence with that of the published genomes of 18 inbred strains (https://www.sanger.ac.uk/resources/mouse/genomes/) (Keane *et al.* 2011) and the male and female C57BL/6J genomes. The ∼40× average coverage of the 2.7 billion base pair reference genome confirms the already known SNPs between C57BL/6J and C57L/J and presents a large number of novel SNPs.

We selected the C57L/J private variants and focused on the ones that had a high impact. Among these is the variant leading to a premature stop codon in *Mlph* (p.R31*), which causes the distinct leaden coat color. Striking in this list of 49 variants is the significant number of genes associated with susceptibility to viral infections (*Klra17*, *H2-D1*, and *H2-T3*). Several of these are within the confidence interval of a QTL for resistance to murine cytomegalovirus in a cross between C57L/J and MA/My (Stadnisky *et al.* 2010). According to a previous study, disruptions in *Ankrd17* are embryonic lethal (Hou *et al.* 2009). Therefore, we were surprised to find a frame shift mutation and a premature stop codon in *Ankrd17*, which one would predict to lead to a similar phenotype, yet C57L/J mice are viable. Another interesting finding is that C57L/J has a unique variant in *Oplah* leading to a frame shift in the C-terminal part of 5-oxoprolinase. Mutations in this gene lead to 5-oxoprolinuria in humans (Calpena *et al.* 2013).

Despite its relatedness to C57BL/6J, the Mouse Phenome Database shows large phenotypic differences between the two strains. For example, both strains are on opposite extreme ends of the strain distribution for plasma sodium levels in 18-month-old female mice. Genetic mapping identified *Nalcn* as a candidate gene underlying this difference (Sinke *et al.* 2011), and comparing the coding sequence shows us a nonsynonymous SNP in exon 44 leading to a p.T1699S amino acid change.

In conclusion, we present a high-quality genome sequence of the C57L/J mouse inbred strain and further expand the number of strains with complete genome sequences. These data allow for better genetic mapping and identification of QTL genes when using the C57L/J strain. In addition, some of the variants unique to C57L/J might identify this strain as a novel model for some human phenotypes, like 5-oxoprolinase and plasma sodium levels.
